# When Being Social Is Costly: Empathy's Role for Risk States, Acute Symptoms, and Cumulative Disease Severity of Depression

**DOI:** 10.1002/jclp.70190

**Published:** 2026-07-28

**Authors:** Dahna Choi, Paula L. Usemann, Katharina Förster, Roland Zahn, Vincent Hammes, Frederike Stein, Florian Thomas‐Odenthal, Lea Teutenberg, Kira Flinkenflügel, Julia Hubbert, Christoph Jurischka, Judith Krieger, Elisabeth J. Leehr, Susanne Meinert, Tim Hahn, Jonathan Repple, Nils Opel, Hamidreza Jamalabadi, Andreas Jansen, Benjamin Straube, Igor Nenadić, Udo Dannlowski, Tilo Kircher, Nina Alexander, Philipp Kanske

**Affiliations:** ^1^ Institute of Clinical Psychology and Psychotherapy TUD Dresden University of Technology Dresden Germany; ^2^ Department of Psychiatry and Psychotherapy Faculty of Medicine Philipps‐Universität Marburg Marburg Germany; ^3^ Center for Mind, Brain and Behavior University of Marburg Marburg Germany; ^4^ Child and Adolescent Psychotherapy, Institute of Psychology, Faculty of Psychology and Movement Science Universität Hamburg Hamburg Germany; ^5^ Department of Psychological Medicine Centre for Affective Disorders, King's College London, Institute of Psychiatry, Psychology & Neuroscience London UK; ^6^ Institute for Translational Psychiatry University of Münster Münster Germany; ^7^ Institute for Translational Neuroscience University of Münster Münster Germany; ^8^ Department of Psychiatry, Psychosomatic Medicine and Psychotherapy University Hospital Frankfurt Goethe University Frankfurt Germany; ^9^ Cooperative Brain Imaging Center ‐ CoBIC Goethe University Frankfurt Frankfurt Germany; ^10^ Department of Psychiatry & Neuroscience Charité University Medicine Berlin Berlin Germany; ^11^ German Centre for Mental Health (DZPG) Berlin Germany; ^12^ Division of Neurology Faculty of Medicine University of British Columbia Vancouver British Columbia Canada; ^13^ Department of Psychology Faculty of Psychology and Educational Sciences Babeş‐Bolyai University Cluj‐Napoca Romania

**Keywords:** empathic concern, empathic distress, major depressive disorder, perspective‐taking, social cognition

## Abstract

**Objectives:**

Patients with major depressive disorder (MDD) frequently report burdensome interpersonal difficulties and altered empathy and perspective‐taking. Previous findings on binary comparisons between patients and healthy controls are limited in their clinical translation as they neglect dissociable risk and disease states. In this preregistered study (https://osf.io/k6mdh/overview?view_only=04b7f39f59a8434292cd472120f661ca), we extend case‐control comparisons by additionally investigating empathic distress, empathic concern, and perspective‐taking in association with (a) familial MDD risk, (b) acute symptom severity, and (c) cumulative MDD severity.

**Method:**

We analyzed data from *n* = 499 MDD patients and *n* = 670 healthy controls from the Marburg‐Münster‐Affective‐Cohort Study. Robust linear regressions were run to investigate group differences in self‐reported empathy and perspective‐taking and their associations with acute symptom severity and familial MDD risk. Analyses on cumulative MDD severity were run in a structural equation model.

**Results:**

Empathic distress was positively associated with both acute and cumulative MDD severity and was elevated in patients compared to healthy controls. A positive association between empathic distress and familial MDD risk became non‐significant after adjustment for age and sex. Patients also reported higher levels of empathic concern—likely influenced by acute symptom severity—and slightly lower levels of perspective‐taking.

**Conclusions:**

Our associational findings indicate empathic distress to constitute a correlate of acute MDD and longer‐term MDD progression, while its relevance for MDD risk requires further investigation. We provide new indications of a generalized heightened emotional attunement during acute depression, entailing both empathic concern and empathic distress. Longitudinal studies are warranted to probe causal associations to prospectively inform psychotherapeutic interventions addressing maladaptive empathic processes.

## 1 Introduction

Humans are inherently social beings, and meaningful interpersonal relationships are considered a basic human need. Difficulties in maintaining these relationships and in navigating social interactions are associated with various mental health conditions, including major depressive disorder (MDD). For example, individuals with MDD often experience social withdrawal, less social engagement, and diminished enjoyment in social interactions (Hirschfeld et al. 2000).

In this study, we elucidate alterations in social affect and social cognition as potential mechanisms underlying interpersonal difficulties in MDD. We distinguish cognitive empathy – also referred to as perspective‐taking or Theory of Mind, involving the ability to reason about and infer others' cognitive or emotional states (Frith and Frith 2005)—and affective empathy, which we define as the shared experience of another's emotions, resulting in an isomorphic emotional state in the observer (De Vignemont and Singer 2006). Affective empathy can have differential outcomes: While it can lead to feelings of warmth and care for another person—termed empathic concern or compassion—it can also induce aversive, self‐oriented feelings of empathic distress (Singer and Klimecki 2014; Trautmann et al. 2022).

The affective and cognitive route of understanding others have shown to be dissociable both behaviorally and neurologically (Kanske et al. 2016; Schurz et al. 2021) and to be differentially impaired in conditions such as autism or psychopathy (Lamm et al. 2016). In MDD, the precise patterns of socio‐affective and ‐cognitive alterations are still to be elucidated. Previous findings indicate impaired perspective‐taking (Bora and Berk 2016; Nestor et al. 2022) and heightened empathic distress in MDD (Banzhaf et al. 2018; Ding et al. 2023; Schreiter et al. 2013). As for empathic concern, chronic depression has been associated with reduced empathic concern for close others (Frick et al. 2021), and compassion‐based interventions have shown symptom‐reducing effects in MDD (Kirby et al. 2017). Contradictory findings of unimpaired social cognition or blunted affective empathy, supposedly resulting from an MDD‐related restricted affective responsiveness, have however also been reported (see e.g., Field et al. 2009; Hoffmann et al. 2016). Drawing clear conclusions based on previous studies is moreover hindered by blurry conceptual notions of social affect and cognition. Although theoretical considerations and preliminary empirical findings give rise to distinguishable associations of empathic distress, empathic concern, and perspective‐taking with depressive symptoms, their deliberate conceptual distinction has been largely neglected (see also Eisenberg et al. 2024).

Another limitation in previous studies comprises the predominant reliance on binary comparisons between MDD patients and healthy individuals. Such approaches overlook the heterogeneity within both patient and healthy samples regarding risk and disorder trajectories. Recent studies indicate the relevance of considering within‐group differences for properly differentiating MDD‐related socio‐affective and ‐cognitive patterns, for example, indicating distinct alterations in empathy depending on developmental stages (Yan et al. 2021) or acute disorder states (Choi et al. 2024).

In the present study, we disentangle socio‐affective and ‐cognitive correlates of MDD both between and within samples of MDD patients and healthy controls (HCs). Within the patient sample, we consider associations with acute as well as cumulative MDD severity, the latter serving as a compound measure including data on the number and duration of depressive episodes, psychiatric hospitalizations, and the time since the first symptom manifestation. Both acute and cumulative MDD severity might significantly but differentially relate to socio‐affective and ‐cognitive alterations, capturing interindividual differences not only based on momentary symptom burden but also based on recurrence of episodes and severity of illness trajectory. A differential predictive value of cumulative disease severity measures compared to momentary symptom measures has been shown for example for neurocognitive decline in MDD (Paolillo et al. 2020).

Regarding indications on longer‐term trends in social affect and cognition in MDD, findings point towards alterations becoming more pronounced with chronicity, with deficits in perspective‐taking posing a risk factor for recurrent depressive episodes (Inoue et al. 2006), higher social distress in chronic versus episodic MDD (Domes et al. 2016), and reduced empathic concern in chronically depressed patients versus HCs (Frick et al. 2021). Therefore, we expect maladaptive socio‐affective and ‐cognitive patterns—in the sense of higher empathic distress, lower empathic concern, and lower perspective‐taking—to be present at acute states of MDD and to be more pronounced in higher cumulative MDD severity. To further probe these patterns as premorbid risk factors of MDD, we investigate their associations with familial MDD risk in HCs. Although a proper differentiation of vulnerability from scarring markers—that is, disorder correlates that show before versus after the first disorder manifestation—requires longitudinal investigations, extending our investigation to risk populations allows to preliminarily explore the onset of socio‐affective and ‐cognitive alterations. Alterations that show during risk states, therefore before a potential prospective disorder manifestation, might be considered for early‐detection screenings and timely prevention (see e.g., Burcusa and Iacono 2007).

In summary, using a large data set of HCs and patients with MDD, we explored self‐reports of empathic distress, empathic concern, and perspective‐taking in (a) group comparisons between MDD patients and HCs, and in association with (b) patients' acute symptom severity, (c) patients' cumulative MDD severity, and (d) familial MDD risk in HCs. For the latter two, we preregistered our analyses and hypothesized both cumulative MDD severity and familial MDD risk to show positive associations with empathic distress, and negative associations with empathic concern and perspective‐taking. Investigations on cumulative MDD severity were run in a structural equation model (SEM) in which we also tested mediation effects between the measures of social affect and cognition (see OSF preregistration: https://osf.io/k6mdh/overview?view_only=04b7f39f59a8434292cd472120f661ca).

## 2 Materials & Methods

### 2.1 Study Design and Sample

Participant data were drawn from the Marburg‐Münster‐Affective‐Cohort‐Study. The data analyzed in this manuscript were derived from the 2‐year follow‐up assessment at which empathy and perspective‐taking were assessed for the first time. Comprehensive study details are described elsewhere (Kircher et al. 2019). Participant recruitment was conducted via newspaper advertisement and flyers and in psychiatric hospitals. General exclusion criteria comprised the following: age < 18 or > 65 years at the time of the initial inclusion into the study, MRI contraindications, history of traumatic brain injury, neurological, cardiovascular, or other severe medical conditions (e.g., cancer, infections, and autoimmune disease), current or lifetime alcohol dependence, current drug dependence or benzodiazepine use, verbal IQ ≤ 80, withdrawal of consent, missing data on clinical diagnoses or non‐European ancestry (to ensure internal validity for genetic and neurobiological analyses that are not relevant for the analysis presented here). Healthy participants were required to have no current or lifetime mental disorder nor any current intake of psychotropic medication. Participants in the MDD sample were permitted to have comorbidities except from the aforementioned alcohol‐ or substance‐related disorders. MDD was required to be the primary diagnosis in all patients, while other comorbidities were permitted. All participants received financial compensation and provided written informed consent prior to participation. The authors assert that all procedures contributing to this work comply with the ethical standards of the relevant national and institutional committees on human experimentation and with the Helsinki Declaration of 1975, as revised in 2013. All procedures involving human subjects were approved by the Ethics Committees of the Medical Faculties of University of Marburg (AZ: 07/14) and University of Münster (2014‐422‐b‐S).

### 2.2 Measured Variables

#### 2.2.1 Sociodemographic and Clinical Assessment

Study participants' sociodemographic data were acquired via paper‐pencil questionnaires. Clinical diagnoses were established with the Structured Clinical Interview I for DSM‐IV (Wittchen et al. 1997), conducted by trained study personnel. Participants' previous disease course was further assessed by the Life Chart method, based on which we obtained data on participants' cumulative MDD severity, including data on the number and cumulative duration of depressive episodes, number and cumulative duration of psychiatric hospitalizations, and time since the first psychiatric symptom manifestation (age at study participation minus age of onset). Acute depressive symptom severity was enquired via self‐reports with the Beck Depression Inventory II (BDI‐II, Beck et al. 1996) and via third‐person ratings from trained personnel with the Hamilton Depression Rating Scale (HAMD, Hamilton 1960).

#### 2.2.2 Familial MDD Risk

Familial MDD risk was assessed via self‐report and cross‐checked in clinical interviews. Participants were categorized as being at familial MDD risk if they reported to have at least one first‐degree relative diagnosed with and treated for MDD.

#### 2.2.3 Social Affect and Social Cognition

As measures of social affect and cognition, data from the 16‐item German version of the Interpersonal Reactivity Index (IRI; Paulus 2009) were considered, which participants filled in as part of a larger online questionnaire battery. The IRI is one of the most commonly used self‐report measures for empathy (Raimondi et al. 2023) and consists of the following subscales: *empathic concern* (feeling of sympathy and concern for others), *personal distress* (self‐oriented feelings of personal anxiety and unease in tense interpersonal settings, subsequently referred to as empathic distress), *perspective‐taking* (tendency to adopt the psychological point of view of others), and *fantasy* (tendency to transpose oneself imaginatively into the feelings and actions of fictitious characters; Davis 1980). In accordance with previous publications from our group (e.g., Jauk et al. 2023), IRI perspective‐taking (α = 0.77) was considered as an indicator of perspective‐taking, and IRI empathic concern (α = 0.65) and IRI empathic distress (α = 0.77) were considered as indicators of empathic concern and empathic distress, respectively.

### 2.3 Statistical Analysis

#### 2.3.1 Between‐Group Analysis

To examine group differences between the HC and MDD sample in empathic distress, empathic concern, and perspective‐taking, we conducted separate robust linear regression analyses. Analyses were run with the MASS package in R (Venables and Ripley 2002). Statistical significance of coefficients was assessed with robust standard errors.

#### 2.3.2 Within‐Group Analysis in the MDD Sample: Acute Symptom Severity

To assess associations between current depressive symptomatology and socio‐affective and ‐cognitive functioning, we conducted robust linear regression analyses with each IRI subscale as the dependent variable. Both self‐reported (BDI‐II; Beck et al. 1996) and clinician‐rated (HAMD; Hamilton 1960) measures of acute symptom severity were considered as predictors in separate models. As above, analyses were conducted using the MASS package in R, with statistical inference based on robust standard errors.

#### 2.3.3 Within‐Group Analysis in the MDD Sample: Cumulative MDD Severity

To investigate the relationship between cumulative MDD severity and socio‐affective and ‐cognitive traits, we employed an SEM approach. Drawing upon previous principal component analyses (Lemke et al. 2022; Zaremba et al. 2018), we defined two latent constructs of cumulative MDD severity: (1) disease duration (comprising the number and total duration of depressive episodes and time since symptom onset), and (2) hospitalization (comprising the number and total duration of psychiatric hospitalizations). By modeling these two factors, we aimed to best fit the data based on previous empirical findings (Lemke et al. 2022; Zaremba et al. 2018), although we did not hypothesize those factors to show diverging associations with the IRI subscales on social affect and cognition. A schematic representation of the SEM including the hypothesized associations with cumulative MDD severity is provided in Supporting Information (Figure D1).

SEM analyses were conducted using the lavaan package in R (version 4.3.1; Rosseel 2012). Because directional hypotheses were not prespecified for all paths in the SEM, we report lavaan's default two‐sided inference consistently throughout. For the prespecified hypotheses, we interpret the relevant SEM paths in light of those a priori predictions. As Mardia's test and Shapiro‐Wilk tests indicated deviations from normality, we used the robust Maximum Likelihood Estimator (MLR), which yields scaled test statistics and robust standard errors. Missing data were addressed via Full Information Maximum Likelihood (FIML), allowing all available data to be retained. To assess the robustness of significant path coefficients, we conducted Scaled Chi‐Square Difference Tests (Satorra and Bentler 2001) to compare the full model against reduced models in which significant paths were constrained to zero (see also Gonzalez and Griffin 2001).

#### 2.3.4 Within‐Group Analysis in the HC Sample: Familial MDD Risk

Due to significant deviations from univariate and multivariate normality of relevant variables according to Mardia's test and Shapiro Wilk test, robust one‐sided Mann‐Whitney u tests were run to investigate whether individuals with versus without elevated familial MDD risk differed in empathic distress, empathic concern, and perspective‐taking. To allow for controlling for potential confounding effects of age and sex, we additionally conducted exploratory robust linear regression analyses, predicting each of the three IRI subscales from MDD risk status (familial MDD risk vs. no risk). Analyses were run with the MASS package in R. Statistical significance of coefficients was assessed with robust standard errors.

### 2.4 Inference Criteria and Confounder Adjustment

Statistical significance was determined using a conventional alpha level of *p* < 0.05. Effect sizes were interpreted based on Cohen's conventions (Cohen 1988). In addition to the unadjusted models as our primary analysis on the crude association of interest, secondary adjusted models were fitted in all our analyses to examine the influence of potential demographic confounding by age and sex (see Eid et al. 2019; Greenberg et al. 2023; McDonald and Kanske 2023).

## 3 Results

### 3.1 Sample Characteristics

The final sample comprised *n* = 499 participants with MDD and *n* = 670 HCs. Participants were aged 20–68 years (*M* = 38.45, SD = 13.46); 64.12% female. Within the MDD sample, 34.7% of participants fulfilled criteria for a current depressive episode at the time of study participation. Lifetime comorbidities were reported in 24.2% of participants (current comorbidities: 13%) and predominantly comprised eating disorders, post‐traumatic stress disorder, and specific phobia (for an overview of lifetime and current comorbidities in the MDD sample see Supporting Information S1: Table A4). Within the HC sample, *n* = 164 reported to have at least one first‐degree relative diagnosed with and treated for MDD, while *n* = 486 did not. There were significant age differences between MDD patients and HCs (*W* = 153418, *p* = 0.016), and between HCs with versus without familial MDD risk (*W* = 44069, *p* = 0.041). The familial MDD risk versus no‐risk samples moreover significantly differed in sex, χ^2^(1) = 10.00, *p* = 0.003. Descriptive statistics of IRI subscales and clinical data on cumulative MDD severity are reported in the Supporting Information S1 (Tables A1–A3, Figures A1–A6).

### 3.2 Group Differences in Social Affect and Cognition

**Figure 1:**
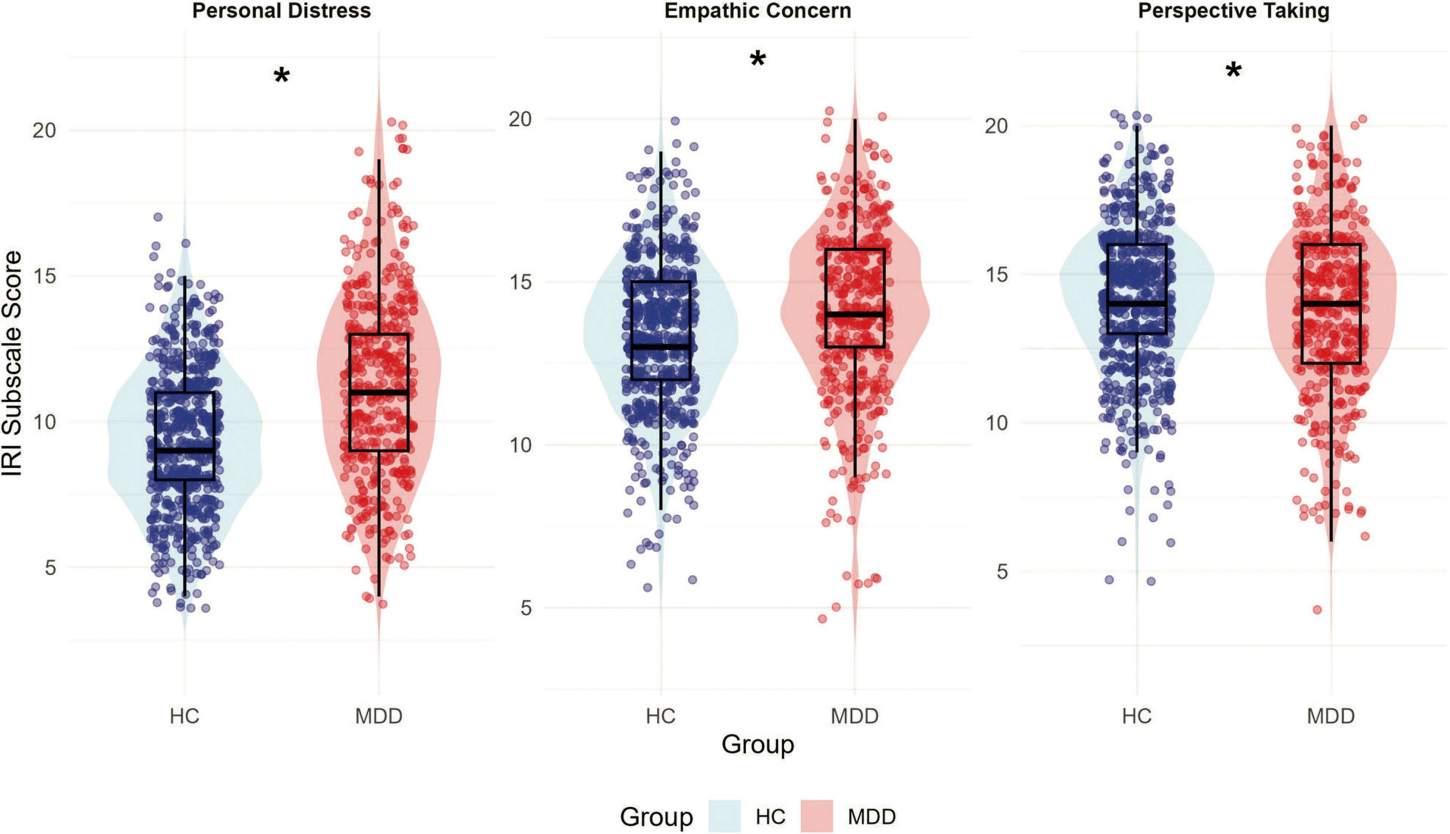
Violin plots comparing IRI subscales (Empathic Distress, Empathic Concern, Perspective‐Taking) between healthy controls (depicted in blue) and individuals with major depressive disorder (depicted in red). HC, healthy controls; IRI, Interpersonal Reactivity Index; MDD, major depressive disorder. *p < 0.05.

Results from robust linear regression analyses without covariates indicate higher levels of empathic distress (β = 1.928, 95% CI [1.562, 2.293], SE = 0.187, *p* < 0.001) and empathic concern (β = 0.761, 95% CI [.479, 1.042], SE = 0.144, *p* < 0.001) in MDD patients compared to HCs, both with large effect sizes. These effects remained stable with age and sex included as covariates. As for perspective‐taking, MDD patients showed lower scores compared to HCs (β = –0.327, 95% CI [−0.647, −0.006], SE = 0.164, *p* = 0.046). This medium‐sized effect became non‐significant when controlling for age and sex (β = –0.282, 95% CI [−0.596, 0.032], SE = 0.160, *p* = 0.079), with age showing a significant negative association with perspective‑taking (see Supporting Information S1: Tables B1–B6 for all regression results on group differences). Histograms of IRI subscales in the MDD versus HC sample are depicted in Figure 1.

### 3.3 Acute Symptom Severity and Social Affect and Cognition

Robust regression analyses within the MDD sample indicated small but significant positive associations between empathic distress and acute depressive symptom severity as measured by both self‐report (BDI‐II: β = 0.135, 95% CI [0.106, 0.163], SE = 0.014, *p* < 0.001) and clinician rating (HAMD: β = 0.218, 95% CI [0.173, 0.262], SE = 0.023, *p* < 0.001). Empathic concern was positively associated with both BDI‐II (β = 0.042, 95% CI [.019, 0.067], SE = 0.012, *p* < 0.001) and HAMD scores (β = 0.074, 95% CI [0.035, 0.113], SE = 0.020, *p* < 0.001), with both effects classified as very small. No significant associations were observed for perspective‐taking. Across all IRI subscales, results remained stable when controlling for age and sex (see Supporting Information S1: Tables C1–C12 and Figures C1–C6 for all results on acute symptom severity).

### 3.4 Cumulative MDD Severity and Social Affect and Cognition

To examine associations with cumulative MDD severity, we used an SEM approach with the MLR estimator due to significant deviations from multivariate (Mardia's skewness = 7981.31, *p* < 0.001; Kurtosis = 222.170) and univariate (all *p* < 0.001) normality. The model showed partial but not conclusive fit. While some fit indices (CFI = 0.91, SRMR = 0.05) were within acceptable ranges, other fit indices (TLI = 0.83, RMSEA = 0.10) indicated poor fit (see Supporting Information S1: Tables D1–D8 for detailed SEM results). The SEM included two latent variables reflecting disease duration (number and duration of depressive episodes and time since first symptom onset) and psychiatric hospitalization (number and duration of psychiatric hospitalizations).

**Figure 2:**
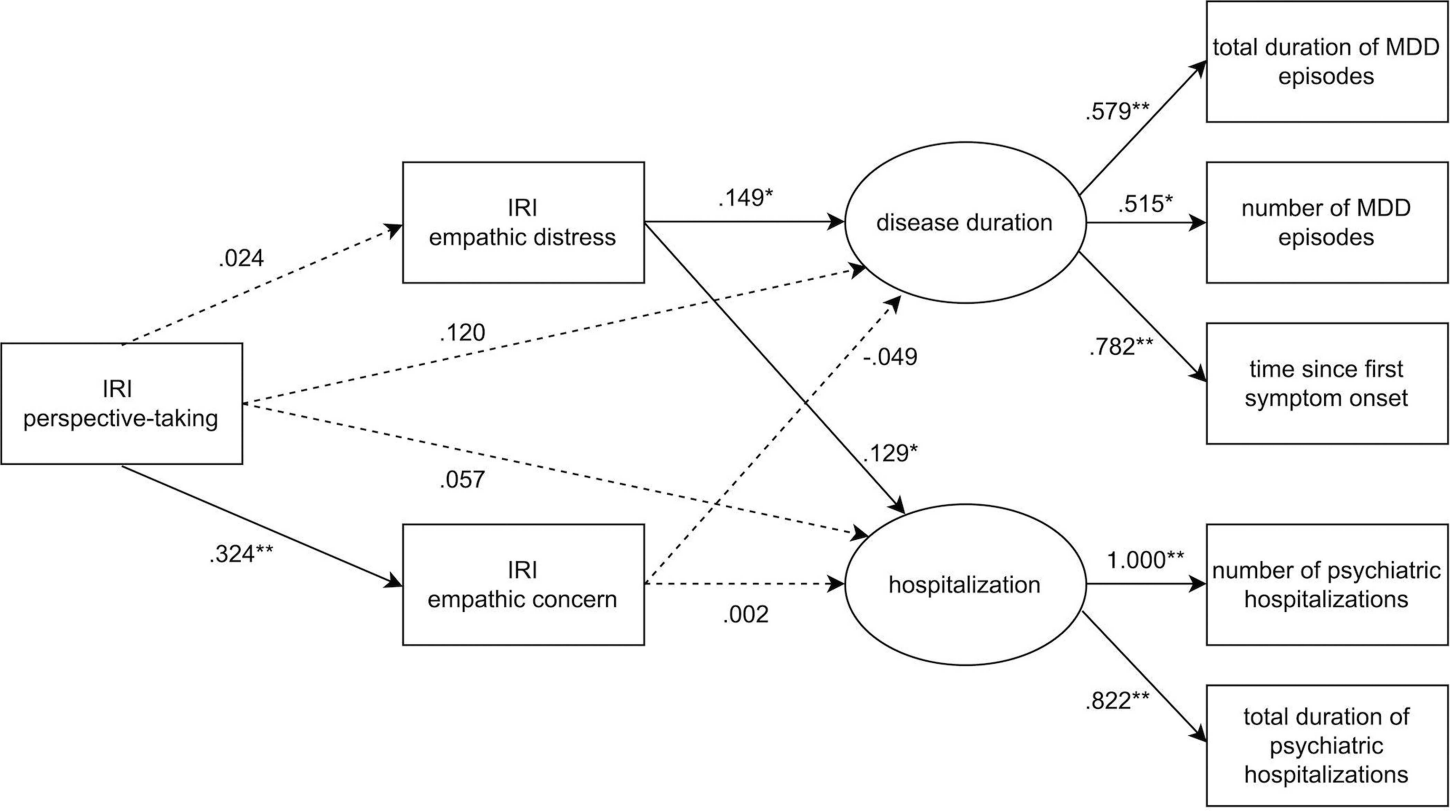
Results from structural equation modeling analyses with standardized path coefficients. Standardized estimates only are reported. Solid lines represent significant paths; dashed lines represent non‐significant paths. IRI, Interpersonal Reactivity Index; MDD, major depressive disorder. *p < 0.05. **p < 0.001.

Empathic distress showed significant associations—although very small in magnitude—with both disease duration (*b* = 1.535, 95% CI [0.166, 2.903], SE = 0.698, *p* = 0.028, β = 0.149) and hospitalization (*b* = 0.077, 95% CI [0.014, 0.140], SE = 0.032, *p* = 0.017, β = 0.129). Perspective‐taking was positively associated with empathic concern (*b* = 0.303, 95% CI [0.213, 0.393], SE = 0.046, *p* < 0.001, β = 0.324), yet none of the other hypothesized direct or mediated associations reached statistical significance. Scaled Chi‐Square Difference Tests supported the robustness of the significant path coefficients (empathic distress → disease duration: Δχ^2^(1) = 7.402, *p* = 0.007; empathic distress → hospitalization: Δχ^2^(1) = 5.876, *p* = 0.015; perspective‐taking → empathic concern: Δχ^2^(1) = 34.559, *p* < 0.001). Figure 2 presents the final SEM model with path coefficients.

### 3.5 Familial MDD Risk and Social Affect and Cognition

**Figure 3:**
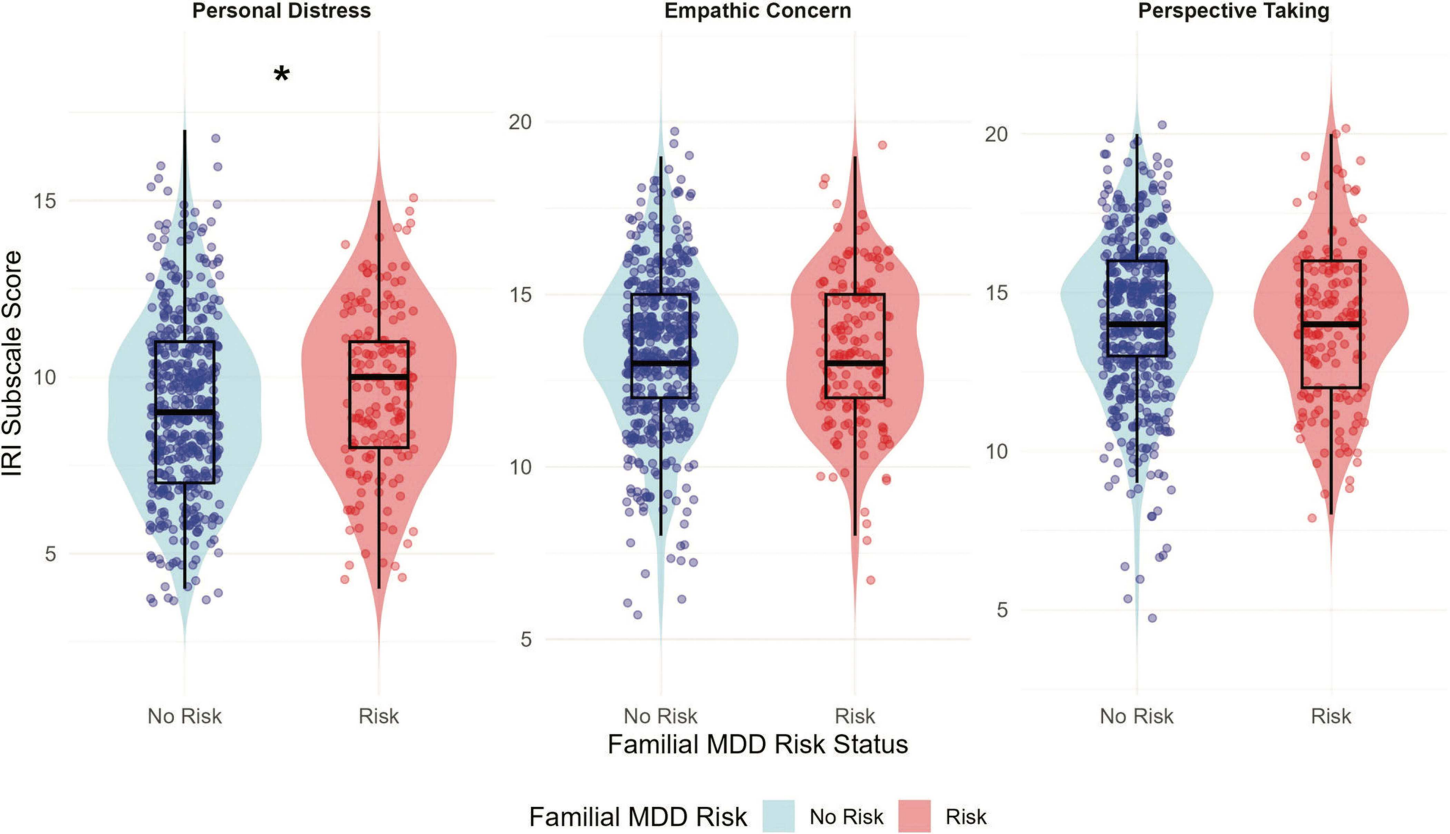
Violin plots of IRI subscales (Empathic Distress, Empathic Concern, Perspective‐Taking) in healthy controls with versus without familial MDD risk. HC, healthy controls; IRI, Interpersonal Reactivity Index; MDD, major depressive disorder. *p < 0.05.

To examine whether altered social affect and cognition might reflect a premorbid vulnerability to MDD, we compared HCs with versus without familial MDD risk. Given deviations from univariate (all *p* < 0.001) and multivariate normality (Mardia's skewness = 34.004; *p* = 0.019; Kurtosis = 2.345), robust Mann‐Whitney u tests were employed. Results showed significantly higher levels of empathic distress in the familial MDD risk group (*W* = 34110, *p* = 0.016). No significant differences were evident for empathic concern or perspective‐taking. Complementary exploratory robust regression analyses without covariates confirmed a positive association between familial MDD risk and empathic distress with medium effect size (β = 0.479, 95% CI [0.049, 0.910], SE = 0.220, *p* = 0.029). When adding age and sex as covariates, sex yielded a significant effect in the sense of higher empathic distress in female participants. In this exploratory adjusted model, the effect of familial MDD risk became non‐significant, though the direction and magnitude remained stable (β = 0.383, 95% CI [−0.062, 0.828], SE = 0.227, *p* = 0.091). All regression results on familial MDD risk are detailed in Supporting Information S1: Tables E1–E11. Histograms for the IRI subscales in HCs with and without familial MDD risk are presented in Figure 3. An overview of all study findings is provided in Table 1.

**Table 1:** Overview of analysis results.

	Empathic distress	Empathic concern	Perspective‐taking
MDD versus HC	MDD > HC	MDD > HC	MDD < HC
Acute symptom severity^a^	↑	↑	n.s.
Cumulative MDD severity	↑	n.s.	n.s.
Familial MDD risk versus no risk	Risk > no risk*	n.s.	n.s.

*Note*: An upward‐pointing arrow (↑) indicates a positive association.

Abbreviations: HC, healthy controls; MDD, major depressive disorder; n.s., non‐significant.

^a^ Results apply for both self‐reported (BDI‐II) and clinician‐rated (HAMD) measures of acute symptom severity.

* Note that the association became non‐significant when adjusting for age and sex.

## 4 Discussion

In this study, we examined socio‐affective and ‐cognitive characteristics associated with depression across different illness states and progressions. Drawing upon a large sample of individuals with current or past MDD and HCs, we analyzed self‐reported empathic distress, empathic concern, and perspective‐taking in relation to group (MDD vs. HC), acute symptom severity, cumulative MDD severity, and familial MDD risk. Based on previous indications, we hypothesized a maladaptive socio‐affective and ‐cognitive profile across MDD risk and manifestation, comprising positive associations with empathic distress and negative associations with empathic concern and perspective‐taking.

Overall, our findings reveal social affect and cognition to be differentially altered in MDD and moreover indicate differential patterns depending on MDD state and progression. First, in group comparisons, MDD patients showed significantly higher levels of both empathic distress and empathic concern compared to healthy individuals. Perspective‐taking was lower in patients, though the effect became non‐significant when controlling for age and sex. Second, both self‐reported and clinician‐rated acute symptom severity showed positive associations with empathic distress and empathic concern and no significant associations with perspective‐taking. Third, supporting our preregistered hypothesis, empathic distress was positively associated with cumulative MDD severity. Contrary to our expectations, no associations emerged between cumulative MDD severity and empathic concern or perspective‐taking. Lastly, also supporting our hypotheses, healthy individuals with compared to those without familial MDD risk reported higher empathic distress, though this association weakened and became non‐significant when controlling for age and sex. We could not find evidence supporting the hypothesized associations of familial MDD risk with empathic concern or perspective‐taking.

### 4.1 Altered Social Affect and Cognition Throughout the Course of MDD

Our differential analysis of empathic distress, empathic concern, and perspective‐taking highlights their dissociable relevance for risk and manifest states of depression. Empathic distress emerged as the most robust correlate of depression, as having shown significant cross‐sectional associations with group status, acute symptom severity, cumulative MDD severity, and familial MDD risk. Regarding the effect of familial MDD risk, it is however to be noted that the effect became non‐significant in the model adjusted for covariates, with female sex showing a positive association with empathic distress. Considering the higher percentage of females in HCs with versus without familial MDD risk, risk‐related elevations in empathic distress might have been confounded by female sex. Therefore, the association between familial MDD risk and empathic distress should be interpreted cautiously. While the effect size remained comparable, it did not remain statistically significant after adjustment for age and sex. Our findings of elevated empathic distress at higher levels of cumulative MDD severity provide indications against the notion of emotional blunting in MDD (e.g., Field et al. 2009). Longitudinal studies are warranted to further probe whether longer‐term disease courses are indeed associated with persistent or even escalating socio‐affective sensitivity. The pronounced relevance of empathic distress compared to other measures of social affect and cognition for longer‐term disease severity corresponds with findings from a previous study where patients with chronic versus episodic MDD showed higher levels of empathic distress and no significant differences in empathic concern nor perspective‐taking (Domes et al. 2016). Together, our indications of elevated empathic distress in association with risk and cumulative MDD severity extend previous indications of bidirectional relationships between depression and affective empathy (Ding et al. 2023). Empathic distress might yield a higher risk for developing MDD, and both acute and longer‐term depressive symptoms might in turn exacerbate empathic distress responses (Thoma et al. 2011).

Importantly, not only empathic distress but also empathic concern was elevated in MDD patients compared to HCs and was positively associated with acute symptom severity. These results contradict previous findings and our hypotheses on empathic concern—as a supposedly more adaptive reaction to others' emotions—to be impaired in MDD (e.g., Frick et al. 2021). In extension to mere group comparisons, our findings open up new considerations of altered social affect depending on specific states of MDD: During acute depressive episodes, emotional attunement may be heightened in a more generalized nature, entailing heightened levels of both empathic distress and empathic concern. This may reflect a non‐specific increase in emotional sensitivity rather than a clear‐cut maladaptive pattern restricted to empathic distress. Considering that empathic concern was however not significantly associated with cumulative MDD severity nor familial MDD risk, elevated empathic concern might be state‐dependent rather than a stable trait marker of depression. Our indications of robustly elevated empathic distress contrary to state‐dependent elevations in empathic concern correspond with previous study results (Müller et al. 2024) and point to a possible dissociation between empathic concern in acute versus chronic manifestations of depressive symptoms. Importantly, it is to be noted that contrary to our hypothesis, empathic concern did not show a negative but positive association with acute symptom severity. While we originally hypothesized empathic concern to be of adaptive value and therefore to be negatively associated with depression, other researchers have suggested lower empathic concern to reflect patients' higher assertiveness after successful psychotherapeutic treatment (Müller et al. 2024). The adaptiveness of empathic concern might therefore require more differential considerations in future investigations. Moreover, given the comparatively lower internal consistency of the empathic concern subscale (α = 0.65), future studies should replicate these findings using measures with greater reliability or complementary behavioral indicators.

Lastly, regarding social cognition, we did not find evidence for the hypothesized negative associations between perspective‐taking and acute symptom severity, cumulative MDD severity, or familial MDD risk. In the binary comparison between patients and healthy controls, perspective‐taking scores were descriptively lower in patients; however, this group difference was no longer significant when age and sex were included as covariates. Considering the significantly higher age in MDD patients compared to HCs, and the negative association between age and perspective‐taking, an age‐related confound in the observed group difference in perspective‐taking cannot be precluded. Together, based on our predominantly non‐significant results on perspective‐taking, we were not able to unravel potential differential alterations in perspective‐taking at different MDD states and progressions. However, rather than interpreting our findings as indications of lacking perspective‐taking alterations in the MDD course, we suggest that non‐significant findings might have been driven by limitations to self‐reports of perspective‐taking. Discrepancies between self‐reported and behavioral measures that have shown to be especially relevant for cognitive compared to affective aspects of empathy (see Murphy and Lilienfeld 2019). Further supporting this notion, previous meta‐analyses with non‐significant results have exclusively focused on self‐report measures of perspective‐taking (Yan et al. 2021), while meta‐analytic results based on task‐based measures of perspective‐taking report significant perspective‐taking deficits in MDD (Bora and Berk 2016; Nestor et al. 2022). Potentially, patients may underestimate the extent of their perspective‐taking difficulties, as social desirability concerns and compensatory efforts to reduce negative self‐perceptions may bias item endorsement independently of actual ability. In contrast to our predominantly non‐significant findings based on self‐reported perspective‐taking, emerging prospective investigations including behavioral or neuroimaging data support the predictive value of impaired social cognition for both MDD onset (see Abraham et al. 2022) and further illness progression (Inoue et al. 2006). Extending this work to additional data modalities and longitudinal designs may therefore yield more valid insights into time‐varying perspective‐taking alterations across the MDD trajectory.

### 4.2 Limitations and Outlook

Regarding our findings on cumulative MDD severity, it should be noted that model fit indices reflected mixed results. While SRMR indicated good fit and CFI was acceptable, TLI and RMSEA suggested inadequate fit, indicating that the current model only partially captures the data structure. Prospective studies might benefit from theoretical model refinement, for example by re‐specifying non‐significant paths or integrating additional clinical data reflecting cumulative MDD severity.

Moreover, MDD risk classification relied on participants' binary self‐reports regarding whether at least one first‐degree relative had been diagnosed with MDD. Although trained interviewers cross‐validated these reports during the clinical interview and the respective relative's treatment for MDD was required for a positive risk classification, residual misclassification cannot be ruled out. Similarly, as for self‐reports of empathy and perspective‐taking, biases related to social desirability and introspective limits may have influenced the data, potentially contributing to discrepancies between participants' reports and their actual socio‐affective and socio‐cognitive dispositions. In addition, as parts of the assessment were conducted online, reduced experimental control and context variability may have further influenced response behavior.

Regarding our findings' clinical translation, it is to be noted that the presence of comorbid conditions in our MDD sample limits conclusions regarding disorder specificity, as the observed associations may reflect depression, comorbidity, or their combined influence. As comorbidities were not explicitly controlled for in the analyses, their potential impact cannot be ruled out. At the same time, including comorbidities enhances ecological validity by better reflecting the clinical reality of MDD. Future studies would benefit from sufficiently powered subgroups to disentangle MDD‐specific effects from broader transdiagnostic influences.

Finally, given that our study sample was derived from a larger study restricted to participants of West‐European ancestry (see Kircher et al. 2019), potential culture‐specific biases in our findings limit their generalizability to non‐western populations. Both the perception of empathy, perspective‐taking, and depressive symptomatology, as well as their specific mutual interdependence can be expected to be subject to cultural imprints (see also Jami et al. 2024; Karasz 2005). Future research should be extended to culturally diverse samples and test whether the observed associations reflect universal or culture‐specific patterns.

Follow‐up investigations should adopt multimodal study designs in order to explore potential discrepancies between self‐reports and actual socio‐affective and ‐cognitive capacities and propensities, for example by the integration of behavioral task‐based or neuroimaging paradigms. Considering the inherently interpersonal nature of empathy and perspective‐taking, prospective integrations of a second‐person perspective might relevantly extend our study findings by approximating real‐life social interaction dynamics and elevate ecological validity. Most importantly, longitudinal study designs are warranted to validate our correlational findings and further elucidate our indications of bidirectional relationships between social affect and cognition and MDD.

## 5 Conclusions

In our study, we have approximated modeling longer‐term developments in MDD‐related social affect and cognition by investigating empathic distress, empathic concern, and perspective‐taking in association with MDD risk states, acute symptom severity, and cumulative MDD severity. Our associational findings indicate elevated empathic distress to be associated with both acute and longer‐term MDD progression, and moreover point towards a potentially generalized heightened affective sensitivity during acute episodes, including elevated levels of empathic concern. Preliminary indications of the presence of elevated empathic distress at MDD risk states require further investigation. We provide crucial support for the notion of empathy as a “risky strength” which should inform psychoeducation of patients, risk cohorts, and their close others. Considering the malleability of empathy and perspective‐taking by training (Förster and Kanske 2021; Trautwein et al. 2020), psychotherapeutic interventions informed by our research are promising approaches for reducing the illness burden of patients and risk cohorts.

## Ethics Statement

The authors assert that all procedures contributing to this work comply with the ethical standards of the relevant national and institutional committees on human experimentation and with the Helsinki Declaration of 1975, as revised in 2013. All procedures involving human subjects were approved by the Ethics Committees of the Medical Faculties of University of Marburg (AZ: 07/14) and University of Münster (2014‐422‐b‐S).

## Conflicts of Interest

J.R. received speaker's honoraria from Janssen, Hexal, Neuraxpharm and Novartis. R.Z. is a private psychiatrist service provider at The London Depression Institute and co‐investigator on a Livanova‐funded observational study of Vagus Nerve Stimulation for Depression. R.Z. has received honoraria for talks at medical symposia sponsored by Lundbeck, Neuraxpharm as well as Janssen. He has collaborated with EMOTRA, EMIS PLC and Depsee Ltd. He is affiliated with the D'Or Institute of Research and Education, Rio de Janeiro and advises the Scients Institute, USA.

## Supporting information


Supporting File

## Data Availability

The data, analytic code, and research material that support the findings of this study are available from the corresponding author, DC, upon reasonable request.
