# Supplementary material for: When Being Social Is Costly: Empathy's Role for Risk States, Acute Symptoms, and Cumulative Disease Severity of Depression
Source: J Clin Psychol. 2026 Jul 28;82(10):1650–60. doi: 10.1002/jclp.70190 (PMC13544485; doi:10.1002/jclp.70190)
Supplement: Supplementary file 1 [file JCLP-82-1650-s001.docx]

**Supplementary Material**

Table of Contents

[Supplement A. Descriptive Statistics of IRI Subscales and Clinical Data in the MDD Sample	5](#_Toc232088405)

[Table A1	5](#_Toc232088406)

[Descriptive Statistics for IRI Subscales by Group (HC vs. MDD)	5](#_Toc232088407)

[Figure A1	6](#_Toc232088408)

[Histogram of IRI Empathic Distress by Group (HC vs. MDD)	6](#_Toc232088409)

[Figure A2	7](#_Toc232088410)

[Histogram of IRI Empathic Concern by Group (HC vs. MDD)	7](#_Toc232088411)

[Figure A3	8](#_Toc232088412)

[Histogram of IRI Perspective-Taking by Group (HC vs. MDD)	8](#_Toc232088413)

[Table A2	9](#_Toc232088414)

[Descriptive Statistics for IRI Subscales in Healthy Controls With versus Without Familial MDD Risk	9](#_Toc232088415)

[Figure A4	10](#_Toc232088416)

[Histogram of IRI Empathic Distress by Familial MDD Risk (Risk vs. no Risk)	10](#_Toc232088417)

[Figure A5	11](#_Toc232088418)

[Histogram of IRI Empathic Concern by Familial MDD Risk (Risk vs. no Risk)	11](#_Toc232088419)

[Figure A6	12](#_Toc232088420)

[Histogram of IRI Perspective-Taking by Familial MDD Risk (Risk vs. no Risk)	12](#_Toc232088421)

[Table A3	13](#_Toc232088422)

[Descriptive Statistics of Variables on Cumulative Disease Severity in Individuals With MDD	13](#_Toc232088423)

[Table A4	14](#_Toc232088424)

[Lifetime and Current Comorbidites in the MDD Sample	14](#_Toc232088425)

[Supplement B: Robust Linear Regression Results on Associations Between Group (HC versus MDD) and Empathic Distress, Empathic Concern, and Perspective-Taking	15](#_Toc232088426)

[Table B1	15](#_Toc232088427)

[Robust Linear Regression Results for Empathic Distress and Group (HC vs. MDD)	15](#_Toc232088428)

[Table B2	16](#_Toc232088429)

[Robust Linear Regression for Empathic Distress and Group (HC vs. MDD) With Covariates of Age and Sex	16](#_Toc232088430)

[Table B3	17](#_Toc232088431)

[Robust Linear Regression for Empathic Concern and Group (HC vs. MDD)	17](#_Toc232088432)

[Table B4	18](#_Toc232088433)

[Robust Linear Regression for Empathic Concern and Group (HC vs. MDD) With Covariates of Age and Sex	18](#_Toc232088434)

[Table B5	19](#_Toc232088435)

[Robust Linear Regression for Perspective-Taking and Group (HC vs. MDD)	19](#_Toc232088436)

[Table B6	20](#_Toc232088437)

[Robust Linear Regression for Perspective-Taking and Group (HC vs. MDD) With Covariates of Age and Sex	20](#_Toc232088438)

[Supplement C: Robust Linear Regression Results on Associations Between Acute Symptom Severity and Empathic Distress, Empathic Concern, and Perspective-Taking	21](#_Toc232088439)

[Table C1	21](#_Toc232088440)

[Robust Linear Regression for Empathic Distress and Self-Reported Acute MDD Symptom Severity	21](#_Toc232088441)

[Table C2	22](#_Toc232088442)

[Robust Linear Regression for Empathic Distress and Self-Reported Acute MDD Symptom Severity with Covariates of Age and Sex	22](#_Toc232088443)

[Figure C1	23](#_Toc232088444)

[Scatterplot of Empathic Distress and Self-Reported Acute MDD Symptom Severity	23](#_Toc232088445)

[Table C3	24](#_Toc232088446)

[Robust Linear Regression for Empathic Concern and Self-Reported Acute MDD Symptom Severity	24](#_Toc232088447)

[Table C4	25](#_Toc232088448)

[Robust Linear Regression for Empathic Concern and Self-Reported Acute MDD Symptom Severity With Covariates of Age and Sex	25](#_Toc232088449)

[Figure C2	26](#_Toc232088450)

[Scatterplot of Empathic Concern and Self-Reported Acute MDD Symptom Severity	26](#_Toc232088451)

[Table C5	27](#_Toc232088452)

[Robust Linear Regression for Perspective-Taking and Self-Reported Acute MDD Symptom Severity	27](#_Toc232088453)

[Table C6	28](#_Toc232088454)

[Robust Linear Regression for Perspective-Taking and Self-Reported Acute MDD Symptom Severity with Covariates of Age and Sex	28](#_Toc232088455)

[Figure C3	29](#_Toc232088456)

[Scatterplot of Perspective-Taking and Self-Reported Acute MDD Symptom Severity	29](#_Toc232088457)

[Table C7	30](#_Toc232088458)

[Robust Linear Regression for Empathic Distress and Clinician-Rated Acute MDD Symptom Severity	30](#_Toc232088459)

[Note. HAMD, Hamilton Depression Rating Scale.	30](#_Toc232088460)

[Table C8	31](#_Toc232088461)

[Robust Linear Regression for Empathic Distress and Clinician-Rated Acute MDD Symptom Severity with Covariates of Age and Sex	31](#_Toc232088462)

[Figure C4	32](#_Toc232088463)

[Scatterplot of Empathic Distress and Clinician-Rated Acute MDD Symptom Severity	32](#_Toc232088464)

[Table C9	33](#_Toc232088465)

[Robust Linear Regression for Empathic Concern and Clinician-Rated Acute MDD Symptom Severity	33](#_Toc232088466)

[Table C10	34](#_Toc232088467)

[Robust Linear Regression for Empathic Concern and Clinician-Rated Acute MDD Symptom Severity with Covariates of Age and Sex	34](#_Toc232088468)

[Figure C5	35](#_Toc232088469)

[Scatterplot of Empathic Concern and Clinician-Rated Acute MDD Symptom Severity	35](#_Toc232088470)

[Table C11	36](#_Toc232088471)

[Robust Linear Regression for Perspective-Taking and Clinician-Rated Acute MDD Symptom Severity	36](#_Toc232088472)

[Table C12	37](#_Toc232088473)

[Robust Linear Regression for Perspective-Taking and Clinician-Rated Acute MDD Symptom Severity with Covariates of Age and Sex	37](#_Toc232088474)

[Figure C6	38](#_Toc232088475)

[Scatterplot of Perspective-Taking and Clinician-Rated Acute MDD Symptom Severity	38](#_Toc232088476)

[Supplement D: SEM Analysis Results on Association Between Cumulative Disease Severity and Empathic Distress, Empathic Concern, and Perspective-Taking	39](#_Toc232088477)

[Figure D1	39](#_Toc232088478)

[Schematic Representation of the Structural Equation Model with the Hypothesized Associations	39](#_Toc232088479)

[Table D1	40](#_Toc232088480)

[Results from Mardia’s Test of Multivariate Normality	40](#_Toc232088481)

[Table D2	41](#_Toc232088482)

[Results from Shapiro Wilk Test of Univariate Normality	41](#_Toc232088483)

[Table D3	42](#_Toc232088484)

[Model Fit Indices from Structural Equation Modeling	42](#_Toc232088485)

[Table D4	43](#_Toc232088486)

[Parameter Estimates from Structural Equation Modeling	43](#_Toc232088487)

[Table D5	46](#_Toc232088488)

[Modification Indices from Structural Equation Modeling	46](#_Toc232088489)

[Table D6	47](#_Toc232088490)

[Chi-Square Difference Test Model Comparison for the Path from Empathic Distress to Disease Duration	47](#_Toc232088491)

[Table D7	48](#_Toc232088492)

[Chi-Square Difference Test Model Comparison for the Path from Empathic Distress to Hospitalization	48](#_Toc232088493)

[Table D8	49](#_Toc232088494)

[Chi-Square Difference Test Model Comparison for the Path from Perspective-Taking to Empathic Concern	49](#_Toc232088495)

[Supplement E: Robust U-Test and Robust Linear Regression Results on Group Comparisons between HCs With Versus Without Familial MDD Risk in Empathic Distress, Empathic Concern, and Perspective-Taking	50](#_Toc232088496)

[Table E1	50](#_Toc232088497)

[Results from Mardia’s Test of Multivariate Normality	50](#_Toc232088498)

[Table E2	51](#_Toc232088499)

[Results from Shapiro Wilk Test of Univariate Normality	51](#_Toc232088500)

[Table E3	52](#_Toc232088501)

[Results from Wilcoxon Rank-Sum Test and Rank-Biserial Correlation Between Empathic Distress and Familial MDD Risk	52](#_Toc232088502)

[Table E4	53](#_Toc232088503)

[Results from Wilcoxon Rank-Sum Test and Rank-Biserial Correlation Between Empathic Concern and Familial MDD Risk	53](#_Toc232088504)

[Table E5	54](#_Toc232088505)

[Results from Wilcoxon Rank-Sum Test and Rank-Biserial Correlation Between Perspective-Taking and Familial MDD Risk	54](#_Toc232088506)

[Table E6	55](#_Toc232088507)

[Robust Linear Regression Results on the Association Between Empathic Distress and Familial MDD Risk	55](#_Toc232088508)

[Table E7	56](#_Toc232088509)

[Robust Linear Regression Results on the Association Between Empathic Distress and Familial MDD Risk with Covariates of Age and Sex	56](#_Toc232088510)

[Table E8	57](#_Toc232088511)

[Robust Linear Regression Results on the Association Between Empathic Concern and Familial MDD Risk	57](#_Toc232088512)

[Table E9	58](#_Toc232088513)

[Robust Linear Regression Results on the Association Between Empathic Concern and Familial MDD Risk with Covariates of Age and Sex	58](#_Toc232088514)

[Table E10	59](#_Toc232088515)

[Robust Linear Regression Results on the Association Between Perspective-Taking and Familial MDD Risk	59](#_Toc232088516)

[Table E11	60](#_Toc232088517)

[Robust Linear Regression Results on the Association Between Perspective-Taking and Familial MDD Risk with Covariates of Age and Sex	60](#_Toc232088518)

# Supplement A. Descriptive Statistics of IRI Subscales and Clinical Data in the MDD Sample

## Table A1

Descriptive Statistics for IRI Subscales by Group (HC vs. MDD)

| IRI Subscale | Group | *M* | *SD* | median | min | max |
| --- | --- | --- | --- | --- | --- | --- |
| Empathic distress | HC | 9.33 | 2.45 | 9.00 | 4.00 | 17.00 |
|  | MDD | 11.29 | 3.16 | 11.00 | 4.00 | 20.00 |
| Empathic concern | HC | 13.35 | 2.28 | 13.00 | 6.00 | 20.00 |
|  | MDD | 14.04 | 2.56 | 14.00 | 5.00 | 20.00 |
| Perspective-taking | HC | 14.12 | 2.49 | 14.00 | 5.00 | 20.00 |
|  | MDD | 13.77 | 2.74 | 14.00 | 4.00 | 20.00 |

*Note.* HC, healthy controls; IRI, Interpersonal Reactivity Index; MDD, major depressive disorder.

## Figure A1

Histogram of IRI Empathic Distress by Group (HC vs. MDD)

*Note.* Data from healthy control subjects are depicted in blue, data from individuals with MDD are depicted in green. HC, healthy controls; IRI, Interpersonal Reactivity Index; MDD, major depressive disorder.

## Figure A2

Histogram of IRI Empathic Concern by Group (HC vs. MDD)

*Note.* Data from healthy control subjects are depicted in blue, data from individuals with MDD are depicted in green. HC, healthy controls; IRI, Interpersonal Reactivity Index; MDD, major depressive disorder.

## Figure A3

Histogram of IRI Perspective-Taking by Group (HC vs. MDD)

*Note.* Data from healthy control subjects are depicted in blue, data from individuals with MDD are depicted in green. HC, healthy controls; IRI, Interpersonal Reactivity Index; MDD, major depressive disorder.

## Table A2

Descriptive Statistics for IRI Subscales in Healthy Controls With versus Without Familial MDD Risk

| IRI Subscale | MDD Risk | *M* | *SD* | median | min | max |
| --- | --- | --- | --- | --- | --- | --- |
| Empathic distress | No Risk | 9.22 | 2.48 | 9.00 | 4.00 | 17.00 |
|  | Risk | 9.63 | 2.35 | 10.00 | 4.00 | 15.00 |
| Empathic concern | No Risk | 13.35 | 2.33 | 13.00 | 6.00 | 20.00 |
|  | Risk | 13.36 | 2.11 | 13.00 | 7.00 | 19.00 |
| Perspective-taking | No Risk | 14.15 | 2.54 | 14.00 | 5.00 | 20.00 |
|  | Risk | 14.05 | 2.37 | 14.00 | 8.00 | 20.00 |

*Note.* HC, healthy controls; IRI, Interpersonal Reactivity Index; MDD, major depressive disorder.

## Figure A4

Histogram of IRI Empathic Distress by Familial MDD Risk (Risk vs. no Risk)

*Note.* Data from healthy control subjects are depicted in blue, data from individuals with MDD are depicted in green. HC, healthy controls; IRI, Interpersonal Reactivity Index; MDD, major depressive disorder.

## Figure A5

Histogram of IRI Empathic Concern by Familial MDD Risk (Risk vs. no Risk)

*Note.* Data from healthy control subjects are depicted in blue, data from individuals with MDD are depicted in green. HC, healthy controls; IRI, Interpersonal Reactivity Index; MDD, major depressive disorder.

## Figure A6

Histogram of IRI Perspective-Taking by Familial MDD Risk (Risk vs. no Risk)

*Note.* Data from healthy control subjects are depicted in blue, data from individuals with MDD are depicted in green. HC, healthy controls; IRI, Interpersonal Reactivity Index; MDD, major depressive disorder.

## Table A3

Descriptive Statistics of Variables on Cumulative Disease Severity in Individuals With MDD

| Variable | *M* | *SD* | Min | Max | Skewness | Kurtosis |
| --- | --- | --- | --- | --- | --- | --- |
| Time since first symptom manifestation (in months) | 43.28 | 59.95 | 1.00 | 555.00 | 4.37 | 27.27 |
| Total number of MDD episodes | 4.44 | 7.31 | 1.00 | 91.00 | 6.87 | 63.76 |
| Total duration of MDD (in months) | 12.71 | 9.91 | 2.00 | 57.00 | 1.64 | 2.83 |
| Total number of psychiatric hospitalizations | 1.68 | 2.36 | 0.00 | 21.00 | 3.23 | 16.50 |
| Total duration of psychiatric hospitalizations (in weeks) | 11.51 | 18.78 | 0.00 | 187.50 | 3.71 | 21.62 |

*Note.* MDD, major depressive disorder.

## Table A4

Lifetime and Current Comorbidites in the MDD Sample

| Diagnosis | Lifetime comorbidity *n* (%) | Current comorbidity *n* (%) |
| --- | --- | --- |
| Any comorbidity (excl. MDD) | 121 (24.2%) | 65 (13%) |
| Eating disorder | 42 (8.4%) | 19 (3.8%) |
| PTSD | 36 (7.2%) | 17 (3.4%) |
| Specific phobia | 27 (5.4%) | 16 (3.2%) |
| Alcohol use disorder | 22 (4.4%) | 6 (1.2%) |
| OCD | 18 (3.6%) | 9 (1.8%) |
| Substance use disorder | 10 (2%) | 5 (1%) |

*Note.* Number and percentage of participants in the MDD sample with comorbidities. Both lifetime comorbidities as well as comorbidities present at the time of study participation are reported. MDD, major depressive disorder.

# Supplement B: Robust Linear Regression Results on Associations Between Group (HC versus MDD) and Empathic Distress, Empathic Concern, and Perspective-Taking

## Table B1

Robust Linear Regression Results for Empathic Distress and Group (HC vs. MDD)

| Predictor | β [95% CIs] | SE | *z* | *p* |
| --- | --- | --- | --- | --- |
| Intercept | 9.29 | 0.10 | 91.33 | <0.001 |
| Group HC vs. MDD | 1.93 [1.562, 2.293] | 0.19 | 10.33 | <0.001 |

*Note.* HC, healthy controls; MDD, major depressive disorder.

## Table B2

Robust Linear Regression for Empathic Distress and Group (HC vs. MDD) With Covariates of Age and Sex

| Predictor | β [95% CIs] | SE | *z* | *p* |
| --- | --- | --- | --- | --- |
| Intercept | 8.69 | 0.29 | 29.93 | <0.001 |
| Group HC vs. MDD | 1.94 [1.575, 2.974] | 0.18 | 10.55 | <0.001 |
| Age | 0.00 [-.018, .007] | 0.01 | -0.82 | 0.412 |
| Sex | 1.24 [.0885, 1.591] | 0.18 | 6.87 | 6.44e-12 |

*Note.* HC, healthy controls; MDD, major depressive disorder.

## Table B3

Robust Linear Regression for Empathic Concern and Group (HC vs. MDD)

| Predictor | β [95% CIs] | SE | *z* | *p* |
| --- | --- | --- | --- | --- |
| Intercept | 13.40 | 0.09 | 148.71 | <0.001 |
| Group HC vs. MDD | 0.76 [.479, 1.042] | 0.14 | 5.30 | 1.175e-07 |

*Note.* HC, healthy controls; MDD, major depressive disorder.

## Table B4

Robust Linear Regression for Empathic Concern and Group (HC vs. MDD) With Covariates of Age and Sex

| Predictor | β [95% CIs] | SE | *z* | *p* |
| --- | --- | --- | --- | --- |
| Intercept | 12.80 | 0.24 | 55.30 | <0.001 |
| Group HC vs. MDD | 0.77 [.489, 1.047] | 0.14 | 5.39 | 6.92e-08 |
| Age | -0.01 [-.018, .002] | 0.01 | -1.56 | 0.1211 |
| Sex | 1.36 [1.063, 1.661] | 0.15 | 9.36 | <0.001 |

*Note.* HC, healthy controls; MDD, major depressive disorder.

## Table B5

Robust Linear Regression for Perspective-Taking and Group (HC vs. MDD)

| Predictor | β [95% CIs] | SE | *z* | *p* |
| --- | --- | --- | --- | --- |
| Intercept | 14.18 | 0.10 | 140.31 | <0.001 |
| Group HC vs. MDD | -0.33 [-.647, -.006] | 0.16 | -2.00 | 0.0457 |

*Note.* HC, healthy controls; MDD, major depressive disorder.

## Table B6

Robust Linear Regression for Perspective-Taking and Group (HC vs. MDD) With Covariates of Age and Sex

| Predictor | β [95% CIs] | SE | *z* | *p* |
| --- | --- | --- | --- | --- |
| Intercept | 14.78 | 0.27 | 55.84 | <0.001 |
| Group HC vs. MDD | -0.28 [-.596, .032] | 0.16 | -1.76 | 0.0788 |
| Age | -0.02 [-.030, -.008] | 0.01 | -3.26 | 0.0011 |
| Sex | 0.16 [-.168, .489] | 0.17 | 0.96 | 0.3379 |

*Note.* HC, healthy controls; MDD, major depressive disorder.

# Supplement C: Robust Linear Regression Results on Associations Between Acute Symptom Severity and Empathic Distress, Empathic Concern, and Perspective-Taking

## Table C1

Robust Linear Regression for Empathic Distress and Self-Reported Acute MDD Symptom Severity

| Predictor | β [95% CIs] | SE | *z* | *p* |
| --- | --- | --- | --- | --- |
| Intercept | 9.89 | 0.19 | 52.00 | <0.001 |
| BDI-II sumscore | 0.13 [.106, .163] | 0.01 | 9.39 | <0.001 |

*Note.* BDI-II, Beck Depression Inventory II.

## Table C2

Robust Linear Regression for Empathic Distress and Self-Reported Acute MDD Symptom Severity with Covariates of Age and Sex

| Predictor | β [95% CIs] | SE | *z* | *p* |
| --- | --- | --- | --- | --- |
| Intercept | 9.38 | 0.45 | 20.70 | <0.001 |
| BDI-II sumscore | 0.13 [.103, .160] | 0.01 | 8.96 | <0.001 |
| Age | -0.01 [-.029, -.014] | 0.01 | -0.68 | 0.4943 |
| Sex | 1.26 [.663, 1.862] | 0.31 | 4.13 | 3.65e-05 |

*Note.* BDI-II, Beck Depression Inventory II.

## Figure C1

Scatterplot of Empathic Distress and Self-Reported Acute MDD Symptom Severity

*Note.* BDI-II, Beck Depression Inventory II.

## Table C3

Robust Linear Regression for Empathic Concern and Self-Reported Acute MDD Symptom Severity

| Predictor | β [95% CIs] | SE | *z* | *p* |
| --- | --- | --- | --- | --- |
| Intercept | 13.74 | 0.16 | 86.36 | <0.001 |
| BDI-II sumscore | 0.04 [.019, .067] | 0.01 | 3.55 | 0.0004 |

*Note.* BDI-II, Beck Depression Inventory II.

## Table C4

Robust Linear Regression for Empathic Concern and Self-Reported Acute MDD Symptom Severity With Covariates of Age and Sex

| Predictor | β [95% CIs] | SE | *z* | *p* |
| --- | --- | --- | --- | --- |
| Intercept | 13.13 | 0.40 | 33.17 | <0.001 |
| BDI-II sumscore | 0.04 [.018, .064] | 0.01 | 3.48 | 0.0005 |
| Age | -0.01 [-.027, .005] | 0.01 | -1.32 | 0.1868 |
| Sex | 1.57 [1.057, 2.081] | 0.26 | 6.00 | 2.009e-09 |

*Note.* BDI-II, Beck Depression Inventory II.

## Figure C2

Scatterplot of Empathic Concern and Self-Reported Acute MDD Symptom Severity

*Note.* BDI-II, Beck Depression Inventory II.

## Table C5

Robust Linear Regression for Perspective-Taking and Self-Reported Acute MDD Symptom Severity

| Predictor | β [95% CIs] | SE | *z* | *p* |
| --- | --- | --- | --- | --- |
| Intercept | 14.01 | 0.17 | 80.88 | <0.001 |
| BDI-II sumscore | -0.02 [-.043, .011] | 0.01 | -1.15 | 0.2503 |

*Note.* BDI-II, Beck Depression Inventory II.

## Table C6

Robust Linear Regression for Perspective-Taking and Self-Reported Acute MDD Symptom Severity with Covariates of Age and Sex

| Predictor | β [95% CIs] | SE | *z* | *p* |
| --- | --- | --- | --- | --- |
| Intercept | 14.80 | 0.43 | 34.65 | <0.001 |
| BDI-II sumscore | -0.02 [-.043, .010] | 0.01 | -1.25 | 0.2131 |
| Age | -0.02 [-.043, .007] | 0.01 | -2.72 | 0.0066 |
| Sex | 0.33 [-.193, .856] | 0.27 | 1.24 | 0.2151 |

*Note.* BDI-II, Beck Depression Inventory II.

## Figure C3

Scatterplot of Perspective-Taking and Self-Reported Acute MDD Symptom Severity

*Note.* BDI-II, Beck Depression Inventory II.

## Table C7

Robust Linear Regression for Empathic Distress and Clinician-Rated Acute MDD Symptom Severity

| Predictor | β [95% CIs] | SE | *z* | *p* |
| --- | --- | --- | --- | --- |
| Intercept | 10.06 | 0.18 | 54.74 | < 2.2e-16 |
| HAMD sumscore | 0.22 [.173, .262] | 0.02 | 9.55 | < 2.2e-16 |

Note. HAMD, Hamilton Depression Rating Scale.

## Table C8

Robust Linear Regression for Empathic Distress and Clinician-Rated Acute MDD Symptom Severity with Covariates of Age and Sex

| Predictor | β [95% CIs] | SE | *z* | *p* |
| --- | --- | --- | --- | --- |
| Intercept | 9.70 | 0.44 | 21.90 | < 2.2e-16 |
| HAMD sumscore | 0.21 [.163, .261] | 0.03 | 8.45 | < 2.2e-16 |
| Age | -0.01 [-.027, .014] | 0.01 | -0.63 | 0.5302 |
| Sex | 1.01 [.443, 1.570] | 0.29 | 3.50 | 0.0005 |

*Note.* HAMD, Hamilton Depression Rating Scale.

## Figure C4

Scatterplot of Empathic Distress and Clinician-Rated Acute MDD Symptom Severity

*Note.* HAMD, Hamilton Depression Rating Scale.

## Table C9

Robust Linear Regression for Empathic Concern and Clinician-Rated Acute MDD Symptom Severity

| Predictor | β [95% CIs] | SE | *z* | *p* |
| --- | --- | --- | --- | --- |
| Intercept | 13.78 | 0.15 | 91.07 | < 2.2e-16 |
| HAMD sumscore | 0.07 [.035, .113] | 0.02 | 3.69 | 0.0002 |

*Note.* HAMD, Hamilton Depression Rating Scale.

## Table C10

Robust Linear Regression for Empathic Concern and Clinician-Rated Acute MDD Symptom Severity with Covariates of Age and Sex

| Predictor | β [95% CIs] | SE | *z* | *p* |
| --- | --- | --- | --- | --- |
| Intercept | 13.26 | 0.39 | 33.78 | < 2.2e-16 |
| HAMD sumscore | 0.06 [.022, .102] | 0.02 | 3.06 | 0.0022 |
| Age | -0.01 [-.028, .004] | 0.01 | -1.43 | 0.1517 |
| Sex | 1.54 [1.016, 2.064] | 0.27 | 5.76 | 8.576e-09 |

*Note.* HAMD, Hamilton Depression Rating Scale.

## Figure C5

Scatterplot of Empathic Concern and Clinician-Rated Acute MDD Symptom Severity

*Note.* HAMD, Hamilton Depression Rating Scale.

## Table C11

Robust Linear Regression for Perspective-Taking and Clinician-Rated Acute MDD Symptom Severity

| Predictor | β [95% CIs] | SE | *z* | *p* |
| --- | --- | --- | --- | --- |
| Intercept | 14.08 | 0.17 | 81.52 | <0.001 |
| HAMD sumscore | -0.04 [-.091, .012] | 0.03 | -1.52 | 0.1296 |

*Note.* HAMD, Hamilton Depression Rating Scale.

## Table C12

Robust Linear Regression for Perspective-Taking and Clinician-Rated Acute MDD Symptom Severity with Covariates of Age and Sex

| Predictor | β [95% CIs] | SE | *z* | *p* |
| --- | --- | --- | --- | --- |
| Intercept | 14.85 | 0.43 | 34.78 | <0.001 |
| HAMD sumscore | -0.04 [-.087, .009] | 0.02 | -1.60 | 0.1099 |
| Age | -0.03 [-.043, -.007] | 0.01 | -2.73 | 0.0062 |
| Sex | 0.36 [-.165, .895] | 0.27 | 1.35 | 0.1774 |

*Note.* HAMD, Hamilton Depression Rating Scale.

## Figure C6

Scatterplot of Perspective-Taking and Clinician-Rated Acute MDD Symptom Severity

*Note.* HAMD, Hamilton Depression Rating Scale.

# Supplement D: SEM Analysis Results on Association Between Cumulative Disease Severity and Empathic Distress, Empathic Concern, and Perspective-Taking

## Figure D1

Schematic Representation of the Structural Equation Model with the Hypothesized Associations

*Note.* Structural equation model depicting hypothesized associations between empathic distress, empathic concern, and perspective-taking and cumulative MDD severity. Rectangles represent observed measured variables. Ellipses denote unobserved latent variables with arrows pointing to the respective indicators. Hypothesized positive and negative associations are indicated with + and - in the regression paths, respectively. IRI, Interpersonal Reactivity Index; MDD, major depressive disorder.

## Table D1

Results from Mardia’s Test of Multivariate Normality

| Test | Statistic | *p* |
| --- | --- | --- |
| Mardia Skewness | 7981.31 | <0.001 |
| Mardia Kurtosis | 222.17 | <0.001 |

## Table D2

Results from Shapiro Wilk Test of Univariate Normality

| Variable | *W* | *p* |
| --- | --- | --- |
| Total duration of MDD | 0.6142 | <0.001 |
| Total number of MDD episodes | 0.3533 | <0.001 |
| Time since first symptom manifestation | 0.8328 | <0.001 |
| Total duration of psychiatric hospitalizations | 0.7841 | <0.001 |
| Total number of psychiatric hospitalizations | 0.6419 | <0.001 |

## Table D3

Model Fit Indices from Structural Equation Modeling

| Fit_Statistic | Value |
| --- | --- |
| Chi-Square | 99.42 |
| Degrees of Freedom | 23.00 |
| *p* | 0.00 |
| CFI | 0.91 |
| TLI | 0.83 |
| RMSEA | 0.10 |
| SRMR | 0.05 |

*Note.* CFI, Comparative Fit Index; RMSEA, Root Mean Square Error of Approximation; SRMR, Standardized Root Mean Square Residual; TLI, Tucker-Lewis Index.

## Table D4

Parameter Estimates from Structural Equation Modeling

| Dependent variable | op | Predictor | Estimate | SE | *p* | Std.all |
| --- | --- | --- | --- | --- | --- | --- |
| Disease duration | =~ | Total duration of MDD | 1.000 | 0.000 |  | 0.579 |
| Disease duration | =~ | Total number of MDD episodes | 0.117 | 0.047 | 0.013 | 0.515 |
| Disease duration | =~ | Time since first symptom manifestation | 0.232 | 0.074 | 0.002 | 0.782 |
| Hospitalization | =~ | Total duration of psychiatric hospitalizations | 1.000 | 0.000 |  | 1.000 |
| Hospitalization | =~ | Total number of psychiatric hospitalizations | 7.890 | 0.871 | 0.000 | 0.822 |
| Disease duration | ~ | Empathic distress | 1.535 | 0.698 | 0.028 | 0.149 |
| Disease duration | ~ | Perspective-taking | 1.456 | 0.788 | 0.065 | 0.120 |
| Disease duration | ~ | Empathic concern | 0.742 | 0.837 | 0.376 | 0.057 |
| Disease duration | ~ | Age | 1.128 | 0.286 | 0.000 | 0.461 |
| Disease duration | ~ | Sex | 0.747 | 4.187 | 0.858 | 0.011 |
| Hospitalization | ~ | Empathic distress | 0.077 | 0.032 | 0.017 | 0.129 |
| Hospitalization | ~ | Perspective-taking | -0.035 | 0.046 | 0.455 | -0.049 |
| Hospitalization | ~ | Empathic concern | 0.001 | 0.043 | 0.978 | 0.002 |
| Hospitalization | ~ | Age | 0.027 | 0.008 | 0.000 | 0.186 |
| Hospitalization | ~ | Sex | -0.224 | 0.204 | 0.272 | -0.056 |
| Empathic distress | ~ | Perspective-taking | 0.028 | 0.069 | 0.687 | 0.024 |
| Empathic distress | ~ | Age | -0.010 | 0.009 | 0.239 | -0.043 |
| Empathic distress | ~ | Sex | 1.501 | 0.251 | 0.000 | 0.225 |
| Empathic concern | ~ | Perspective-taking | 0.303 | 0.046 | 0.000 | 0.324 |
| Empathic concern | ~ | Age | -0.010 | 0.009 | 0.239 | -0.054 |
| Empathic concern | ~ | Sex | 1.501 | 0.251 | 0.000 | 0.283 |

*Note.* Estimate = unstandardized estimate; op = operator; Std.all = fully standardized estimate based on standardization of all variables.

## Table D5

Modification Indices from Structural Equation Modeling

| Relationship | mi | epc | sepc.all |
| --- | --- | --- | --- |
| Hospitalization =~ Total duration of psychiatric hospitalizations | 15.48 | 6.83 | 0.23 |
| Hospitalization =~ Time since first symptom manifestation | 13.44 | -1.31 | -0.26 |
| Time since first symptom manifestation ~~ Total duration of psychiatric hospitalizations | 17.77 | -2.04 | -10.70 |
| Empathic distress ~~ Empathic concern | 35.86 | 2.20 | 0.32 |
| Empathic distress ~ Empathic concern | 31.77 | 0.40 | 0.32 |
| Empathic concern ~ Hospitalization | 20.65 | 1.76 | 1.32 |
| Empathic concern ~ Empathic distress | 36.20 | 0.23 | 0.29 |

*Note.* epc, expected parameter change; mi, modification index; sepc.all, standardized expected parameter change with all variables standardized.

## Table D6

Chi-Square Difference Test Model Comparison for the Path from Empathic Distress to Disease Duration

| Model | Df | AIC | BIC | Chi_Square | Chisq_Diff | Df_Diff | *p* |
| --- | --- | --- | --- | --- | --- | --- | --- |
| model_fit1 | 23.00 | 16,021.00 | 16,122.00 | 99.42 |  |  |  |
| model_fit2 | 24.00 | 16,025.00 | 16,121.00 | 104.98 | 7.40 | 1.00 | 0.007 |

## Table D7

Chi-Square Difference Test Model Comparison for the Path from Empathic Distress to Hospitalization

| Model | Df | AIC | BIC | Chi_Square | Chisq_Diff | Df_Diff | *p* |
| --- | --- | --- | --- | --- | --- | --- | --- |
| model_fit1 | 23.00 | 16,021.00 | 16,122.00 | 99.42 |  |  |  |
| model_fit2 | 24.00 | 16,024.00 | 16,121.00 | 104.58 | 5.88 | 1.00 | 0.015 |

## Table D8

Chi-Square Difference Test Model Comparison for the Path from Perspective-Taking to Empathic Concern

| Model | Df | AIC | BIC | Chi_Square | Chisq_Diff | Df_Diff | *p* |
| --- | --- | --- | --- | --- | --- | --- | --- |
| model_fit1 | 23.00 | 16,021.00 | 16,122.00 | 99.42 |  |  |  |
| model_fit2 | 24.00 | 16,062.00 | 16,158.00 | 141.91 | 34.56 | 1.00 | <0.001 |

# Supplement E: Robust U-Test and Robust Linear Regression Results on Group Comparisons between HCs With Versus Without Familial MDD Risk in Empathic Distress, Empathic Concern, and Perspective-Taking

## Table E1

Results from Mardia’s Test of Multivariate Normality

| Test | Statistic | *p* |
| --- | --- | --- |
| Mardia Skewness | 34.00 | <0.001 |
| Mardia Kurtosis | 2.35 | 0.019 |

## Table E2

Results from Shapiro Wilk Test of Univariate Normality

| Variable | *W* | *p* |
| --- | --- | --- |
| Empathic distress | 0.9813 | <0.001 |
| Empathic concern | 0.9785 | <0.001 |
| Perspective-taking | 0.9769 | <0.001 |

## Table E3

Results from Wilcoxon Rank-Sum Test and Rank-Biserial Correlation Between Empathic Distress and Familial MDD Risk

| Statistic | Value |
| --- | --- |
| *W* | 34,110.00 |
| *p* | 0.02 |
| Rank-Biserial Correlation | -0.14 |

## Table E4

Results from Wilcoxon Rank-Sum Test and Rank-Biserial Correlation Between Empathic Concern and Familial MDD Risk

| Statistic | Value |
| --- | --- |
| *W* | 38,807.00 |
| *p* | 0.415 |
| Rank-Biserial Correlation | -0.03 |

## Table E5

Results from Wilcoxon Rank-Sum Test and Rank-Biserial Correlation Between Perspective-Taking and Familial MDD Risk

| Statistic | Value |
| --- | --- |
| *W* | 39,786.00 |
| *p* | 0.24 |
| Rank-Biserial Correlation | 0.00 |

## Table E6

Robust Linear Regression Results on the Association Between Empathic Distress and Familial MDD Risk

| Predictor | β [95% CIs] | SE | *z* | *p* |
| --- | --- | --- | --- | --- |
| Intercept | 9.17 | 0.12 | 76.89 | <0.001 |
| factor(Familial MDD risk vs. no risk) | 0.48 [.049, .909] | 0.22 | 2.18 | 0.0291 |

*Note.* MDD, major depressive disorder.

## Table E7

Robust Linear Regression Results on the Association Between Empathic Distress and Familial MDD Risk with Covariates of Age and Sex

| Predictor | β [95% CIs] | SE | *z* | *p* |
| --- | --- | --- | --- | --- |
| Intercept | 8.61 | 0.32 | 26.52 | <0.001 |
| factor(Familial MDD risk vs. no risk) | 0.38 [-.062, .828] | 0.23 | 1.69 | 0.0914 |
| Age | 0.00 [-.018, .011] | 0.01 | -0.50 | 0.6181 |
| Sex | 1.10 [.691, 1.519] | 0.21 | 5.23 | 1.66e-07 |

*Note.* MDD, major depressive disorder.

## Table E8

Robust Linear Regression Results on the Association Between Empathic Concern and Familial MDD Risk

| Predictor | β [95% CIs] | SE | *z* | *p* |
| --- | --- | --- | --- | --- |
| Intercept | 13.41 | 0.11 | 125.29 | <0.001 |
| factor(Familial MDD risk vs. no risk) | -0.04 [-.424, .344] | 0.20 | -0.20 | 0.8387 |

*Note.* MDD, major depressive disorder.

## Table E9

Robust Linear Regression Results on the Association Between Empathic Concern and Familial MDD Risk with Covariates of Age and Sex

| Predictor | β [95% CIs] | SE | *z* | *p* |
| --- | --- | --- | --- | --- |
| Intercept | 12.90 | 0.29 | 45.01 | <0.001 |
| factor(Familial MDD risk vs. no risk) | -0.20 [-.579, .171] | 0.19 | -1.06 | 0.2869 |
| Age | -0.01 [-.019, .006] | 0.01 | -0.99 | 0.3212 |
| Sex | 1.21 [.841, 1.572] | 0.19 | 6.47 | <0.001 |

*Note.* MDD, major depressive disorder.

## Table E10

Robust Linear Regression Results on the Association Between Perspective-Taking and Familial MDD Risk

| Predictor | β [95% CIs] | SE | *z* | *p* |
| --- | --- | --- | --- | --- |
| Intercept | 14.22 | 0.12 | 121.49 | <0.001 |
| factor(Familial MDD risk vs. no risk) | -0.19 [-.624, .253] | 0.22 | -0.83 | 0.4061 |

*Note.* MDD, major depressive disorder.

## Table E11

Robust Linear Regression Results on the Association Between Perspective-Taking and Familial MDD Risk with Covariates of Age and Sex

| Predictor | β [95% CIs] | SE | *z* | *p* |
| --- | --- | --- | --- | --- |
| Intercept | 14.76 | 0.34 | 43.04 | <0.001 |
| factor(Familial MDD risk vs. no risk) | -0.21 [-.652, .227] | 0.22 | -0.95 | 0.3432 |
| Age | -0.01 [-.029, -.001] | 0.01 | -2.09 | 0.0364 |
| Sex | 0.06 [-.355, .484] | 0.21 | 0.30 | 0.7636 |

*Note.* MDD, major depressive disorder.
